# Suppressive Effects of Flavonoids on Macrophage-Associated Adipocyte Inflammation in a Differentiated Murine Preadipocyte 3T3-L1 Cells Co-Cultured with a Murine Macrophage RAW264.7 Cells

**DOI:** 10.3390/plants11243552

**Published:** 2022-12-16

**Authors:** Dahae Lee, Sukyong Hong, Kiwon Jung, Sungyoul Choi, Ki Sung Kang

**Affiliations:** 1 Department of Preventive Medicine, College of Korean Medicine, Gachon University, Seongnam 13120, Republic of Korea; 2 College of Pharmacy, CHA University, Sungnam 13844, Republic of Korea; 3 Oncobix Co., Ltd., Yongin-si 16950, Republic of Korea; 4 Department of Neuropsychiatry, College of Korean Medicine, Gachon University, Seongnam 13120, Republic of Korea

**Keywords:** flavonoid, adipocytes, macrophages, adipogenesis, inflammation

## Abstract

The suppressive effects of flavonoids on macrophage-associated adipocyte inflammation in a differentiated murine preadipocyte cell line (3T3-L1) co-cultured with a murine macrophage cell line (RAW264.7) were evaluated. Extracellular lipid accumulation was investigated via Oil Red O staining. The expression levels of adipogenesis- and inflammation-associated proteins, including CCAAT/enhancer-binding protein (C/EBP)-α, inducible nitric oxide synthase (iNOS), C/EBPβ, peroxisome proliferator-activated receptor γ (PPARγ), and cyclooxygenase-2 (COX-2), were determined via Western blotting. Proinflammatory cytokines, including monocyte chemoattractant protein 1 (MCP-1) and interleukin-6 (IL-6), were assessed using enzyme-linked immunosorbent assay kits. We found that silybin, formononetin, and diosmetin inhibited lipid accumulation and production of proinflammatory cytokines in the co-cultures of 3T3-L1 and RAW264.7 cells. Moreover, they inhibited the protein expression of PPARγ, C/EBPα, COX-2, C/EBPβ, and iNOS in the co-cultures of 3T3-L1 and RAW264.7 cells. These data support that silybin, formononetin, and diosmetin inhibit macrophage-associated adipocyte inflammation and lipid accumulation.

## 1. Introduction

Excessive fat accumulation is closely related to an increased hazard of various metabolic diseases, that is, type II diabetes, inflammation, heart disease, and hypertension [1]. Chronic inflammation in adipose tissue, characterized by the disturbed infiltration of macrophages and secretion of proinflammatory cytokines, is related to obesity [2]. The step of differentiation of preadipocytes into adipocytes starts with the exponential growth phase of adipoblasts [3]. Cells go through a limited number of cell divisions to enter cell cycle arrest and then re-enter the cell cycle. In the final stage, they differentiate into mature adipocytes [4]. Adipogenesis and adipocyte hypertrophy induces inflammatory signaling [5]. The macrophage-associated adipocyte inflammation is a risk factor for obesity-related metabolic disorders, which are caused by the accumulation of macrophages in adipose tissue [6]. 

Previous studies have revealed the promising effects of plant-derived flavonoids in treating macrophage-associated adipocyte inflammation due to their ability to inhibit adipogenesis via the reduction of lipid accumulation as well as their anti-inflammatory effects [7,8]. Flavonoids, including silybin [9,10], taxifolin [11,12], myricetin [13,14], quercetin [15,16], baicalein [17,18], formononetin [19,20], genistin [21,22], catechin [23,24], kaempferol [25,26], puerarin [27,28], rutin [29,30], naringin [31,32], and apigenin [26,33], inhibit adipogenesis in murine 3T3-L1 preadipocyte cell lines and inflammation in murine RAW264.7 macrophages; however, not enough is known about their roles using the co-cultures of 3T3-L1 and RAW264.7 cells. In general, co-culture systems are more advantageous than monolayer cell cultures for studying cell-cell interactions in a cellular environment where multiple cells interact with each other [34]. An aglycone part of the flavonoid glycoside, diosmetin, inhibits inflammatory mediators, such as tumor necrosis factor-α (TNF-α), MCP-1, and inducible nitric oxide synthase (iNOS) in the co-cultures of RAW264.7 cells and 3T3-L1 cells [35]. However, not enough is known about the effects of diosmetin on the lipid accumulation and expression of adipogenesis-associated proteins in the co-cultures of RAW264.7 cells and 3T3-L1 cells.

In this experiment, the effects of various flavonoids, including silybin present in *Silybum marianum*, taxifolin present in *Silybum marianum*, diosmetin present in *Caucasian vetch*, myricetin present in *Camellia sinensis* L., quercetin present in *Allium cepa*, phloretin present in *Malus domestica*, baicalein present in the roots of *Scutellaria baicalensis* Georgi, formononetin present in *Phaseolus vulgaris*, genistin present in *Glycine max*, catechin present in *Thea chinensis*, kaempferol present in *Ginkgo biloba*, puerarin present in *Pueraria montana*, rutin present in *Fagopyrum esculentum*, naringin present in *Podocarpus fasciculus*, apigenin present in *Citrus × sinensis*, quercetin 3,7,4′-trimethyl ether (Q1), quercetin 5-methyl ether (Q2), quercetin 7-methyl ether (Q3), quercetin 7,4′-dimethyl ether (Q5), and quercetin pentamethyl ether (Q10) on the inflammatory responses and lipid accumulation in RAW264.7 and 3T3-L1 cells were compared. Additionally, the underlying mechanisms were studied by selecting the silybin, formononetin, and diosmetin that showed significant effects.

## 2. Results

### 2.1. Inhibitory Effects of Flavonoids on Adipogenesis and Lipid Accumulation in Co-Cultures of 3T3-L1 and RAW264.7 Cells

**Figure 1:**
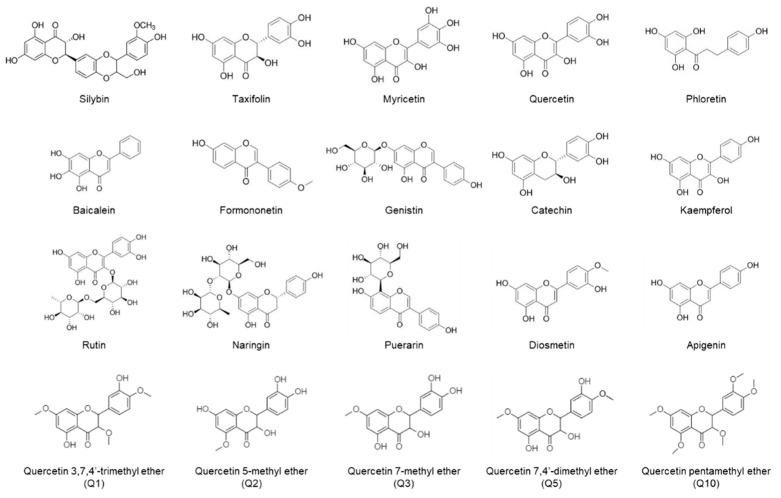
Structures of flavonoids.

**Figure 2:**
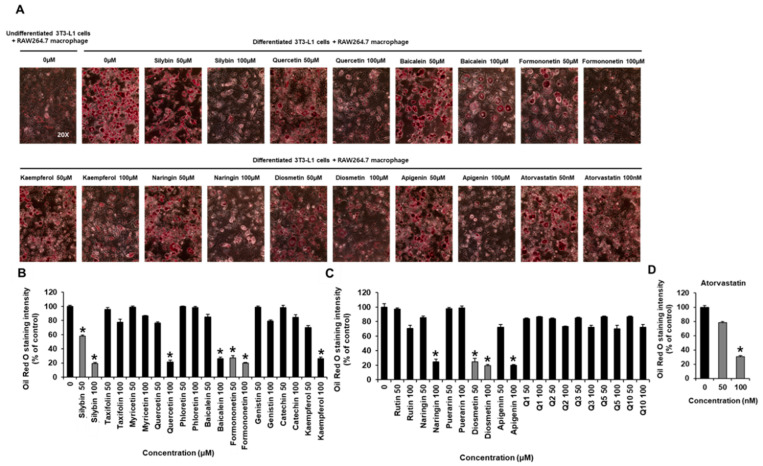
Inhibitory effects of flavonoids and atorvastatin (positive control) on lipid accumulation in the co-cultures of 3T3-L1 and RAW264.7 cells. (**A**) Images and (**B**–**D**) quantification of the Oil Red O stained droplets (absorbance at 490 nm) of the co-cultures of 3T3-L1 and RAW264.7 cells treated with the indicated concentration of flavonoids. In individual experiments, each treatment condition was set up in quadruplicate, and each experiment was repeated three times independently (* *p* < 0.05 compared to untreated cells, Kruskal–Wallis non-parametric test). Data are represented as the mean ± SEM.

The cytotoxicity of various flavonoids, including a flavonolignan (silybin), flavononols (taxifolin, myricetin, quercetin, phloretin, catechin, kaempferol, rutin, Q1, Q2, Q3, Q5, and Q10), flavones (diosmetin, baicalein, naringin, apigenin), and isoflavones (formononetin, genistin, puerarin) was not detected in RAW264.7 cells and 3T3-L1 preadipocytes (Figure 1). Cell viability assay exhibited that flavonoids up to a concentration of 100 µM did not affect the viability of two cell lines (Supplementary Figure S1). Thus, flavonoids at 50 and 100 μM were chosen for subsequent experiments on the co-cultures of RAW264.7 and 3T3-L1 cells. In co-cultures of RAW264.7 and 3T3-L1 cells, silybin (50 and 100 μM), quercetin (100 μM), formononetin (50 and 100 μM), kaempferol (100 μM), naringin (100 μM), diosmetin (50 and 100 μM), apigenin (100 μM), and atorvastatin (100 nM, positive control) inhibited adipogenesis and lipid accumulation performed by Oil Red O staining (Figure 2). Moreover, silybin, formononetin, and diosmetin significantly inhibited Oil Red O staining in a concentration-dependent manner. Thus, silybin, formononetin, and diosmetin were selected for subsequent experiments.

### 2.2. Inhibitory Effects of Flavonoids on the Production of IL-6 and MCP-1 in Co-Cultures of 3T3-L1 and RAW264.7 Cells

**Figure 3:**
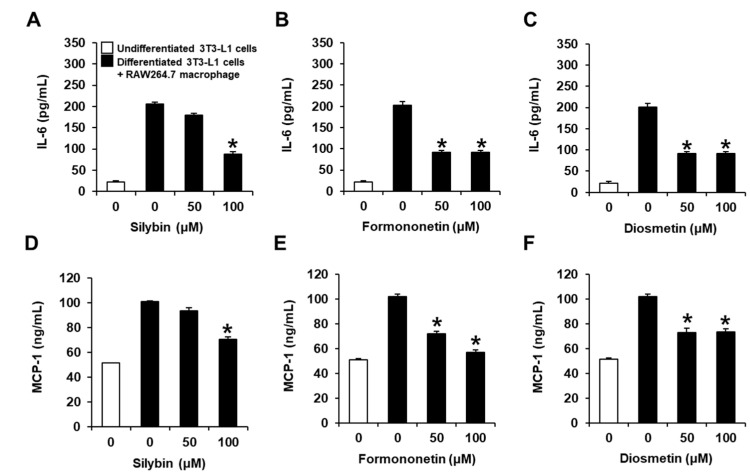
Inhibitory effects of silybin, formononetin, and diosmetin on the production of interleukin (IL)-6 and monocyte chemoattractant protein (MCP)-1 in co-cultures of 3T3-L1 and RAW264.7 cells. Analysis of the proinflammatory cytokine IL-6 production in co-cultures of 3T3-L1 and RAW264.7 cells treated with the indicated concentrations of (**A**) silybin, (**B**) formononetin, and (**C**) diosmetin. Analysis of the proinflammatory cytokine MCP-1 production in co-cultures of 3T3-L1 and RAW264.7 cells treated with the indicated concentrations of (**D**) silybin, (**E**) formononetin, and (**F**) diosmetin. In individual experiments, each treatment condition was set up in quadruplicate, and each experiment was repeated three times independently (* *p* < 0.05 compared to untreated cells, Kruskal–Wallis non-parametric test). Data are represented as the mean ± SEM.

Silybin, formononetin, and diosmetin reduced the production of IL-6 and MCP-1 compared with the co-culture control (Figure 3). These results suggest that silybin (100 μM), formononetin (50 and 100 μM), and diosmetin (50 and 100 μM) inhibit proinflammatory cytokine production in the co-culture system.

### 2.3. Inhibitory Effects of Flavonoids on the Adipogenesis- and Inflammation-Associated Proteins in Co-Cultures of 3T3-L1 and RAW264.7 Cells

**Figure 4:**
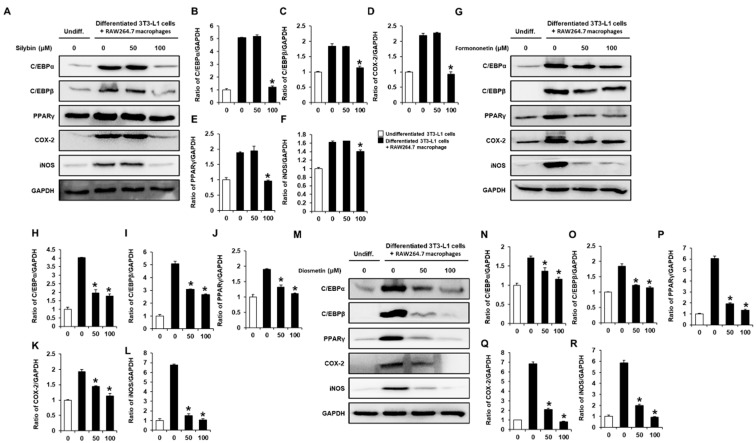
Inhibitory effects of silybin, formononetin, and diosmetin on the adipogenesis- and inflammation-associated proteins in co-cultures of 3T3-L1 and RAW264.7 cells. (**A**–**F**) Protein expression levels and ratios of band intensities of CCAAT/enhancer-binding protein alpha (C/EBPα), C/EBPβ, peroxisome proliferator-activated receptor γ (PPARγ), cyclooxygenase-2 (COX-2), and inducible nitric oxide synthase (iNOS) in co-cultures of 3T3-L1 and RAW264.7 cells treated with the indicated concentration of silybin. (**G**–**L**) Protein expression levels and ratios of band intensities of C/EBPα, C/EBPβ, PPARγ, COX-2, and iNOS in co-cultures of 3T3-L1 and RAW264.7 cells treated with the indicated concentration of formononetin. (**M**–**R**) Protein expression levels and ratios of band intensities of C/EBPα, C/EBPβ, PPARγ, COX-2, and iNOS in co-cultures of 3T3-L1 and RAW264.7 cells treated with the indicated concentration of diosmetin. Undiff, undifferentiated 3T3-L1 cells. In individual experiments, each treatment condition was set up in quadruplicate, and each experiment was repeated 3 times independently (* *p* < 0.05 compared to untreated cells, Kruskal–Wallis non-parametric test). Data are represented as the mean ± SEM.

Consistent with the inhibition of extracellular lipid accumulation and proinflammatory cytokines, silybin (100 μM), formononetin (50 and 100 μM), and diosmetin (50 and 100 μM) decreased the protein expression levels of peroxisome proliferator-activated receptor-γ (PPARγ), CCAAT/enhancer-binding protein (C/EBP)-α, C/EBPβ, inducible nitric oxide synthase (iNOS), and cyclooxygenase-2 (COX-2) (Figure 4). These results indicate that silybin, formononetin, and diosmetin inhibit adipogenesis-associated proteins (PPARγ, C/EBPα, and C/EBPβ) and inflammation-associated proteins (iNOS and COX-2), thus, silybin, formononetin, and diosmetin may ameliorate inflammatory crosstalk between macrophages and adipocytes.

## 3. Discussion

We compared the effects of taxifolin, myricetin, quercetin, phloretin, baicalein, genistin, catechin, kaempferol, puerarin, rutin, naringin, apigenin, Q1, Q2, Q3, Q5, and Q10 on lipid accumulation, as well as silybin, diosmetin, formononetin on the inflammatory responses and lipid accumulation, in RAW264.7 and 3T3-L1 cells. Silybin, quercetin, formononetin, kaempferol, naringin, diosmetin, and apigenin inhibited adipogenesis and lipid accumulation in the co-cultures of RAW264.7 and 3T3-L1 cells. 

A polyphenolic flavonoid, silybin, was obtained from the milk thistle, *Silybum marianum* (L.). The pharmacological activities of silybin have been extensively studied. Its hepatoprotective effects are associated with antioxidant and anti-inflammatory effects [36]. The isoflavone formononetin, found in *Astragalus membranaceus*, exerts anti-obesity, anti-inflammatory, and anti-cancer effects [19,20,37]. Diosmetin inhibits lipolysis and inflammatory mediators, including TNF-α, MCP-1, iNOS, and, NO in the co-cultures of RAW264.7 and 3T3-L1 cells [35]. However, the effects of silybin, formononetin, and diosmetin on the lipid accumulation and expression of adipogenesis-associated proteins in the co-cultures of RAW264.7 and 3T3-L1 cells remain unknown.

In this experiment, consistent with the inhibitory effects of silybin, formononetin, and diosmetin on lipid accumulation, silybin (100 μM), formononetin (50 and 100 μM), and diosmetin (50 and 100 μM) inhibited the levels of proinflammatory cytokines (MCP-1 and IL-6) and inducible enzymes (COX-2 and iNOS), which produce NO that causes inflammation in co-cultures of RAW264.7 and 3T3-L1 cells. It is assumed that 3T3-L1 cells participate in inflammatory crosstalk upon cell-cell contact with RAW264.7 macrophages. These inhibitory effects were also observed by increased levels of proinflammatory cytokines and inducible enzymes, as reported in previous studies [35,38,39]. These results indicate that silybin, formononetin, and diosmetin inhibit macrophage-associated adipocyte inflammation. In addition, silybin, formononetin, and diosmetin inhibited the protein expression levels of adipogenesis-associated proteins (C/EBPβ, C/EBPα, and PPARγ) in co-cultures of RAW264.7 and 3T3-L1 cells. Among PPARs, PPARγ is predominately expressed in adipose tissue and has been known as a critical modulator of adipogenesis. In the intracellular molecular pathways involved in adipogenesis, C/EBPβ activates the transcription of master regulators, such as PPARγ and C/EBPα, which together enhance lipid accumulation and adipogenesis [40]. Inhibition of C/EBPα by silybin was reported by Ka et al. [41], but its effects on PPARγ and C/EBPβ were not identified yet. The inhibitory effect of diosmetin on inflammation and lipolysis in the co-culture of adipocytes and macrophages was recently reported by Lee et al. [35], but the involvement of PPARγ was not identified. However, formononetin exerted PPARγ-driven reporter-gene activity and induced differentiation of 3T3-L1 preadipocytes [42]. This difference is thought to be due to the difference in the experimental conditions and concentration of the formononetin, which is different from our results. 

All flavonoids have two six-membered rings and one three-carbon unit linked through the linear three-carbon chain [43,44]. The glycosides are usually attached at position 3 or 7, and the most common carbohydrates are arabinose, L-rhamnose, galactose, gluco-rhamnose, or D-glucose [45]. However, glycosides had little effect in comparison to the effects of the flavonoids used in this study. Therefore, the effect of aglycone without sugar was good, and the relationship with efficacy can be confirmed through structural comparison of aglycones. The other factors related to the various chemical properties of flavonoids include their hydroxylation patterns, methoxy groups, and conjugation between aromatic rings [45]. Diosmetin is an O-methylated flavone is also known as 5,7,3′-trihydroxy-4′-methoxyflavone. Apigenin is a trihydroxyflavone that is flavone substituted by hydroxy groups at positions 4′, 5 and 7. As a result of comparing diosmetine, which showed the best effect, and apigenin, which had a similar structure but had no effect, it was confirmed that 4′-methoxy was an important structure to exert the effect. Formononetin (7-hydroxy-4′-methoxyisoflavone) is an O-methylated isoflavone. Formononetin was difficult to compare its effect because it did not have a similar structure among the tested compounds, but similarly to diosmetin, 4′-methoxy group seem to be important. However, the 7-hydoxyl group contained in both flavonoids cannot be ignored. It is necessary to further investigate which receptors these flavonoids act on. 

In conclusion, our results revealed that silybin, formononetin, and diosmetin inhibited the inflammatory responses stimulated by the interaction of RAW264.7 and 3T3-L1 cells via downregulating the inflammatory biomarkers. These results can be used to develop potential strategies to alleviate and prevent obesity-related inflammatory disorders. However, further evidence for this result should be evaluated in animal models of obesity. 

## 4. Materials and Methods

### 4.1. Materials

All flavonoids (silybin, taxifolin, diosmetin, myricetin, quercetin, phloretin, baicalein, formononetin, genistin, catechin, kaempferol, puerarin, rutin, naringin, apigenin, Q1, Q2, Q3, Q5, and Q10) and atorvastatin were obtained from Sigma-Aldrich (St. Louis, MO, USA).

### 4.2. Cell Culture

The murine 3T3-L1 preadipocyte cell line (CL-173) and murine RAW264.7-macrophage cell line (TIB-71) were obtained from the American Type Culture Collection (Manassas, VA, USA). The 3T3-L1 preadipocytes were cultured in Dulbecco’s modified Eagle’s medium (DMEM; Cellgro, Manassas, VA, USA) containing 10% fetal calf serum, 25 mmol/L glucose, and 1% penicillin/streptomycin (P/S; Invitrogen Co., Grand Island, NY, USA) in a humidified atmosphere of 5% CO_2_ at 37 °C and 95% air. Under the same conditions, RAW264.7 cells were grown in DMEM containing 10% fetal bovine serum, 25 mmol/L glucose, and 1% P/S (Invitrogen Co).

### 4.3. Cell Viability Assay

The cytotoxicity of 3T3-L1 preadipocytes and RAW264.7 cells was evaluated using an Ez-Cytox cell viability assay kit (Daeil Lab Service Co., Seoul, Republic of Korea). The two cell lines were seeded and incubated with flavonoids (100 μM) for 24 h. After treating the two cell lines with 10% (v/v) Ez-Cytox reagent at 37 °C for 1 h, the viability of the two cell lines was assessed via measuring the optical density (450 nm) using a microplate reader (PowerWave XS; Bio-Tek Instruments, Winooski, VT, USA).

### 4.4. Adipogenic Differentiation Assay and Co-Culture

After reaching confluence, 3T3-L1 preadipocytes were stimulated with insulin (5 µg/mL), dexamethasone (1 µg/mL), and 1-isobutyl-3-methylxanthine (0.5 mM) for 48 h. Then, insulin (5 µg/mL) was added into the cell culture medium, which was replaced with a fresh medium containing insulin (5 µg/mL) every two days until day 8. On day 8, differentiated adipocytes were co-cultured with RAW264.7 cells in the contact system.

### 4.5. Oil Red O Staining

Differentiated 3T3-L1 adipocytes co-cultured with RAW264.7 cells were fixed with paraformaldehyde solution (4%) and stained with Oil Red O solution (0.3%) at room temperature for 1 h. After extraction with isopropanol (100%), the absorbance (490 nm) of the stained oil droplets (red color) was determined using a microplate reader (PowerWave XS).

### 4.6. Measurement of Proinflammatory Cytokine Levels

The levels of MCP-1 and IL-6 in the cell culture supernatant were measured using ELISA kits (R&D Systems, Minneapolis, MN, USA), according to the manufacturer’s protocol. 

### 4.7. Western Blotting Analysis

Cells were lysed using the radioimmunoprecipitation assay buffer (pH 7.4; Cell Signaling Technology, Danvers, MA, USA), protein per lane (20 μg) was separated via 10% sodium dodecyl sulfate-polyacrylamide gel electrophoresis and transferred onto polyvinylidene difluoride membranes. Membranes were incubated with primary antibodies (1:1000 dilution; Cell Signaling Technology) against iNOS (# 13120S), C/EBPα (# 2295S), PPARγ (# 2435S), COX-2 (# 12282S), C/EBPβ (# 43095S), GAPDH (# 2118S) overnight at 4 °C, followed by incubation with secondary antibodies (1:2000 dilution; Cell Signaling Technology). The membranes were treated with the ECL Plus Western Blotting Detection Reagent (GE Healthcare, Little Chalfont, UK) and visualized using a chemiluminescence system (FUSION Solo; PEQLAB Biotechnologie GmbH, Erlangen, Germany). 

### 4.8. Statistical Analysis

All experiments were performed in triplicate. All analyses were performed using SPSS Statistics ver. 19.0 (SPSS Inc., Chicago, IL, USA). Non-parametric comparisons of samples were conducted using the Kruskal–Wallis test to analyze the results. Differences were considered statistically significant at *p* < 0.05.

## 5. Conclusions

Silybin, formononetin, and diosmetin suppressed lipid accumulation and production of IL-6 and MCP-1 in the co-cultures of RAW264.7 and 3T3-L1 cells. They also exerted suppressive effects on adipogenesis- and inflammation-associated proteins, including C/EBPβ, COX-2, C/EBPα, PPARγ, and iNOS, in the co-cultures of RAW264.7 and 3T3-L1 cells. This comparative study indicates that silybin, formononetin, and diosmetin may prevent obesity by suppressing macrophage-associated adipocyte inflammation.

## Supplementary Materials

The following supporting information can be downloaded at https://www.mdpi.com/article/10.3390/plants11243552/s1, Figure S1. Effect of the flavonoids on the viability of 3T3-L1 preadipocytes and RAW264.7 cells. (A and B) Effect of flavonoids compared with the control (0 µM) on the viability of 3T3-L1 preadipocytes for 24 h. Cell viability was determined by the MTT assay. In individual experiments, each treatment condition was set up in quadruplicate, and each experiment was repeated 3 times independently (*p* < 0.05 compared to untreated cells, Kruskal–Wallis non-parametric test). Data are represented as the mean ± SEM.
