# Supplementary material for: Suppressive Effects of Flavonoids on Macrophage-Associated Adipocyte Inflammation in a Differentiated Murine Preadipocyte 3T3-L1 Cells Co-Cultured with a Murine Macrophage RAW264.7 Cells
Source: Plants (Basel). 2022 Dec 16;11(24):3552. doi: 10.3390/plants11243552 (PMC9783032; doi:10.3390/plants11243552)
Supplement: Supplementary file 1 [file plants-11-03552-s001.zip › plants-2071265-supplementary.pdf]

Suppressive effects of flavonoids on macrophage-associated adipocyte inflammation in a differentiated murine preadipocyte 3T3-L1 cells co-cultured with a murine macrophage RAW264.7 cells

Dahae Lee [1], Sukyong Hong [2], Kiwon Jung [2,3], Sung youl Choi [4,*], Ki Sung Kang [1,*]

[1]Department of Preventive Medicine, College of Korean Medicine, Gachon University, Seongnam 13120, Korea; pjsldh@gachon.ac.kr (D. L.); kkang@gachon.ac.kr (K. S. K.)

[2]College of Pharmacy, CHA University, Sungnam 13844, Republic of Korea; hongsk@chauniv.ac.kr (S. H.)

[3]Oncobix Co., Ltd., Yongin-si 16950, Gyeonggi-do, Republic of Korea; pharmj@oncobix.com (K. J.)

[4]Department of Neuropsychiatry, College of Korean Medicine, Gachon University

*Correspondence: pc1075@gachon.ac.kr (S. C.), Tel.: +82-32-770-1343 (S. C.); kkang@gachon.ac.kr (K. S. K.), Tel.: +82-31-750-5402

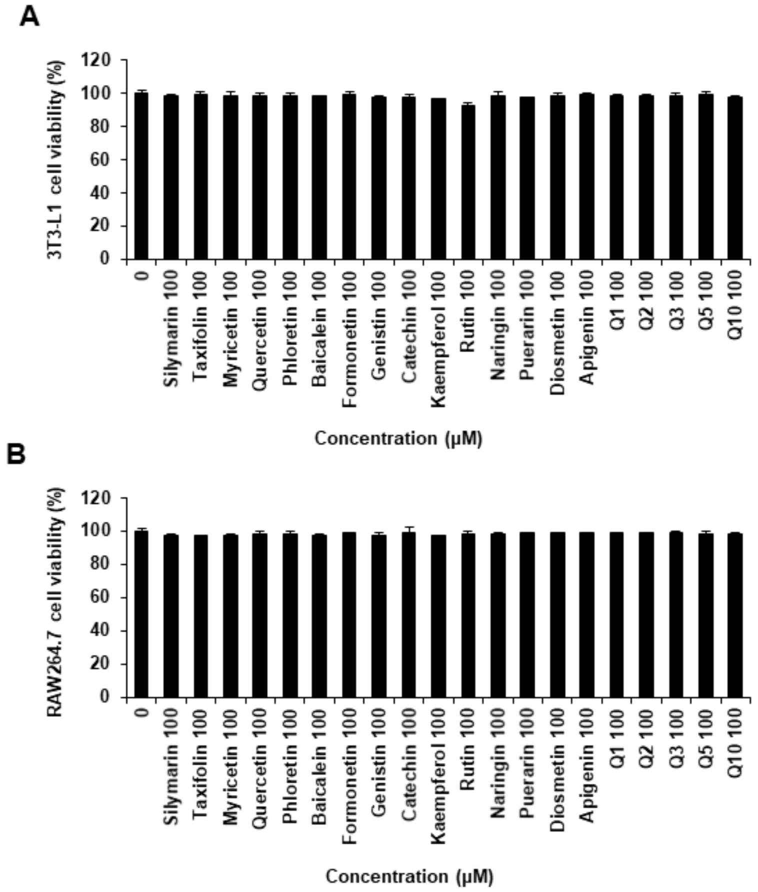

Figure S1. Effect of the flavonoids on the viability of 3T3-L1 preadipocytes and RAW264.7 cells. (A and B) Effect of flavonoids compared with the control (0 μM) on the viability of 3T3-L1 preadipocytes for 24 h. Cell viability was determined by the MTT assay. In individual experiments, each treatment condition was set up in quadruplicate, and each experiment was repeated 3 times independently ($p < 0.05$ compared to untreated cells, Kruskal–Wallis non-parametric test). Data are represented as the mean ± SEM.
